# Supplementary material for: The genome sequence of the White-barred Knot-horn, Elegia similella (Zincken, 1818)
Source: Wellcome Open Res. 2024 Apr 12;9:186. [Version 1] doi: 10.12688/wellcomeopenres.21213.1 (PMC11531641; doi:10.12688/wellcomeopenres.21213.1)
Supplement: Peer review history

# Reviewer report: Reviewer response for version 1

**DOI:** 10.21956/wellcomeopenres.23460.r104972

**Published:** 2024-11-02

**Authors:** Panagiotis Ioannidis

**Affiliations:** 1 Foundation for Research & Technology - Hellas, Crete, Greece

This manuscript presents the sequencing, assembly and annotation of a lepidopteran insect. The quality of the genome assembly is very good (chromosome level).

 I have to say that I'm happy with the fact that the genome assemblies published in this journal, now include a predicted gene set. However, it is absolutely necessary that the authors also measure the quality of this gene using BUSCO. It is very important for whoever wants to use this genome to know how good this gene set is, both in absolute terms (e.g. a gene set having 70% complete BUSCOs can't be very good), as well as in relative terms (i.e. how good the gene set is, compared to the corresponding genome assembly). I would suggest that the authors run BUSCO (using the same lineage they used for the evaluation of the genome assembly) and add the BUSCO scores in the manuscript (Table 1 seems the most fitting place).

 An additional point has to do with the number of chromosomes. The authors say that they have assembled 30 chromosomes, but it would be nice to add whether this is expected. For example, is the number of chromosomes known for other closely related Lepidoptera?

Are sufficient details of methods and materials provided to allow replication by others?

Yes

Is the rationale for creating the dataset(s) clearly described?

Yes

Are the datasets clearly presented in a useable and accessible format?

Yes

Are the protocols appropriate and is the work technically sound?

Yes

Reviewer Expertise:

Insect genomics

I confirm that I have read this submission and believe that I have an appropriate level of expertise to confirm that it is of an acceptable scientific standard.

# Reviewer report: Reviewer response for version 1

**DOI:** 10.21956/wellcomeopenres.23460.r104971

**Published:** 2024-11-01

**Authors:** Bryan Brunet

**Affiliations:** 1 Ottawa Research and Development Centre, Ontario, Canada

The manuscript entitled “The genome sequence of the White-barred Knot-horn, Elegia similella (Zincken, 1818)” by Hammond *et al.* presents a chromosomal genome assembly for *Elegia similella* from a single male individual originating from the UK. The authors use a combination of Pacific Biosciences HiFi long reads and Hi-C data to assemble 30 chromosome-scale scaffolds for this species at a total genome size of 780.4 Mb. The data were also used to recover and assemble the mitogenome. The genome has very good assembly metrics, and all molecular and most bioinformatic methods employed have been sufficiently detailed either in the manuscript itself or with appropriate references to methods described in other publications. Aside from a few minor criticisms noted below, the genome should be a useful contribution to the field and is of scientific soundness and rigor.

- The authors present a photograph of the specimen in Figure 1. It is unknown whether this is sufficient to diagnose the accuracy of the identification, but at very least the authors should identify where morphological vouchers (if any) remain and/or have been deposited so that identification can be confirmed should it be necessary.
- Manual curation steps are described but the precise corrections that were made are not detailed in the manuscript. Such information should be made available in order to allow for repeatability of these steps.
- Annotation was completed using BRAKER2 in protein mode but no information is provided on what proteomic data and/or taxa were used to make these predictions. It would be useful to know the taxonomic extent for the set proteins used for annotation.

Are sufficient details of methods and materials provided to allow replication by others?

Partly

Is the rationale for creating the dataset(s) clearly described?

Yes

Are the datasets clearly presented in a useable and accessible format?

Yes

Are the protocols appropriate and is the work technically sound?

Yes

Reviewer Expertise:

I am an aphid systematist with expertise in genomic approaches in the context of phylogenetics and population genetics. I've been involved in several genome assembly projects.

I confirm that I have read this submission and believe that I have an appropriate level of expertise to confirm that it is of an acceptable scientific standard.

# Reviewer report: Reviewer response for version 1

**DOI:** 10.21956/wellcomeopenres.23460.r100551

**Published:** 2024-10-08

**Authors:** Jeffrey Marcus, Nataliia Kopchak

**Affiliations:** 1 University of Manitoba (Ringgold ID: 124615), Winnipeg, Manitoba, Canada

In this manuscript, the author describes the sequencing and assembly of the *Elegia* *similella* genome using DNA from an adult male specimen collected in the UK. The primary genome sequence assembly includes proposed chromosomal pseudomolecule sequences for 29 autosomes, the Z sex chromosome, and a complete mitochondrial genome. On the whole, this is a useful contribution to the scientific literature, but please see our comments below, especially regarding the title, background, identification of the specimen, and details of mitogenome assembly.

 Some suggestions to the author:

1. **Title: **The title indicates the species' genome sequence, which implies the complete genome, including that autosomes and both Z and W sex chromosomes are being analyzed. However, the paper only discusses the Z chromosome data and doesn't include an analysis of the W chromosome from females. To name the title more accurately with the content, one might write that the study focuses specifically on the male genome. We also note that in the metadata found at https://links.tol.sanger.ac.uk/spe- cies/1101167 , the sex of the specimen is listed as “not collected”, which appears to be incorrect.
2. **Background:** We recommend extending background information about the species (morphology, ecology, distribution, larval host plants, etc.) to provide more context about *Elegia similella*. This will help readers unfamiliar with the species better understand this organism.
3. **Method of Specimen identification: **The specimen identification was named, but which keys/species descriptions were consulted and the morphological characters used for the identification have not been included in the manuscript.
4. **Figure #1:** It would be useful to add a scale bar on the photo, to better understand the size of the organism.
5. The author wrote: “Manual assembly curation corrected 4 missing joins or mis-joins and removed 4 haplotypic duplications, reducing the assembly length….” It would be beneficial to describe how 4 missing joins or mis-joins were identified and how they were corrected.
6. The authors describe how “The mitochondrial genome was assembled using MitoHiFi (Uliano-Silva et al. 2022) which runs MitoFinder (Allio et al. 2020) or MITOS (Bernt et al. 2013) and uses these annotations to select the final mitochondrial contig…”. The authors do not describe which of the algorithms generated the selected contig for the mitogenome.
7. **Genome Annotation:**  In addition to the total number of protein-coding genes, additional description of the preliminary details of the number/proportion of novel genes identified through this analysis should be included in the text of the manuscript.
8. **Reference sequence:** Any reference genomes or sequences used in this genome assembly (for example for the mitochondrial genome assembly) should be described in the main text of the manuscript and formally cited.

Are sufficient details of methods and materials provided to allow replication by others?

Partly

Is the rationale for creating the dataset(s) clearly described?

Partly

Are the datasets clearly presented in a useable and accessible format?

Yes

Are the protocols appropriate and is the work technically sound?

Yes

Reviewer Expertise:

Evolutionary biology of insects, phylogenomics

We confirm that we have read this submission and believe that we have an appropriate level of expertise to confirm that it is of an acceptable scientific standard, however we have significant reservations, as outlined above.
